# Relationship Between Parental Rejection and Problematic Mobile Phone Use in Chinese University Students: Mediating Roles of Perceived Discrimination and School Engagement

**DOI:** 10.3389/fpsyg.2019.00428

**Published:** 2019-03-05

**Authors:** Jianjun Zhu, Ruiqin Xie, Yuanyuan Chen, Wei Zhang

**Affiliations:** School of Psychology, South China Normal University, Guangzhou, China

**Keywords:** parental rejection, perceived discrimination, school engagement, problematic mobile phone use, sequential mediating effect

## Abstract

In order to clarify the onset mechanism of problematic mobile phone use, and to develop better strategies to prevent and treat problematic mobile phone use, the current study tested the negative impact of parental rejection on problematic mobile phone use and the mediating roles of perceived discrimination and school engagement in this association. The sample consisted of 356 Chinese university students (36.3% male) ranging from 17 to 19 years of age. Participants completed self-report questionnaires assessing parental rejection, perceived discrimination, school engagement, and problematic mobile phone use. The results documented that parental rejection was a direct risk factor for problematic mobile phone use. This association was mediated by perceived discrimination, and there was also a sequential mediating effect in which perceived discrimination led in turn to low school engagement.

## Introduction

The use of mobile phones has dramatically increased over the last decades across the world. Among adults, 90% in the United States and 93% in the United Kingdom own a mobile phone (PewResearch, 2014; OFCOM, 2016). In China, as of January 2018, about 1.4 billion mobile phone subscriptions had been registered (Statista, 2018), with young adults (aged 18–22 years) being one of the largest and fastest-growing populations of mobile phone users (Chen et al., 2016). However, whereas the mobile phone brings great convenience for the purposes of communication and entertainment, research suggests that problematic mobile phone use is associated with a range of deficits in physical, psychological, and social functioning (e.g., alcohol abuse, anxiety, low academic performance, and addictive social media use; Ha et al., 2008; Sánchez-Martínez and Otero, 2009; Li et al., 2015).

Problematic mobile phone use in its extreme form is considered a form of behavioral addiction including the core components of addictive behaviors, such as cognitive salience, loss of control, mood modification, tolerance, withdrawal, conflict, and relapse (Billieux et al., 2015). Thus, it is important to pay more attention to problematic mobile phone use and its influences and effects. In the current study, problematic mobile phone use is defined as physiological and psychological discomfort due to inappropriate or excessive use of mobile phones (Xiong et al., 2012). In order to better clarify effective strategies for prevention and intervention, we tested the negative impact of parental rejection on problematic mobile phone use, and the indirect role of perceived discrimination and school engagement in this association, in a sample of Chinese university students.

### Parental Rejection and Problematic Mobile Phone Use

Parental rejection refers to the absence or significant withdrawal of the warmth, affection, care, comfort, concern, nurturance, support, or simply love that individuals optimally receive from their parents and other caregivers, and by the presence of a variety of physically and psychologically hurtful behavior and negative affect (Rohner et al., 2005). Several studies have reported the negative impact of parental rejection on depression (Magaro and Weisz, 2006; Xiao et al., 2017), substance use (Stover and Kiselica, 2015; Stogner and Gibson, 2016), and externalizing behaviors (Daganzo et al., 2014; Putnick et al., 2015; Nawaz et al., 2017) across development. However, the association between parental rejection and problematic mobile phone use among late adolescents remains unclear.

According to problem behavior theory (Jessor, 1987), inappropriate parental supervision, rejection, and lack of affection could damage individual perceptions of their environment and then increase the possibility of problem behaviors (e.g., problematic mobile phone use). A small number of studies have highlighted parenting as an influence on mobile phone addiction (Bae, 2015; Deng et al., 2015; Lian et al., 2016). For instance, a longitudinal study including 2218 early adolescents in South Korea reported that lower authoritative parenting was associated with more addictive use of mobile phones (Bae, 2015). Similarly, Lian et al. (2016) showed that negative parenting style significantly increased the severity of problematic mobile phone use in university students. In this context, it is reasonable to hypothesize that ongoing parental rejection experienced by university students would be positively associated with problematic mobile phone use.

### Perceived Discrimination as the Mediator

Perceived discrimination refers to the individual’s perception of being the target of others’ negative attitudes and unfair treatment (Pascoe and Smart Richman, 2009). In this study, we examined the indirect role of perceived discrimination in the association between parental rejection and problematic mobile phone use. Previous research revealed that high parental rejection was significantly correlated with higher attachment anxiety (Grossmann et al., 2005; Hinnen et al., 2009; Pepping et al., 2015). In accordance with attachment theory (Bowlby, 1969) and parental acceptance–rejection theory (Rohner, 2004), individuals with attachment anxiety are more prone to show negative self-cognition and to use hyperactive strategies in response to stress, which leads them to pay more attention to negative signals and to perceive more discrimination. The positive association between attachment anxiety and perceived discrimination has been reported in earlier research (Zakalik and Wei, 2006).

Additionally, perceived discrimination as an important stressor may lead to problematic mobile phone use. When people perceive that they are discriminated against, they feel more stressed, eliciting a series of stress responses that broadly lead to problem behaviors (Lazarus and Folkman, 1984; Pascoe and Smart Richman, 2009). Previous studies have demonstrated that people sometimes select problematic mobile phone use as a way to cope with stress (Chiu, 2014; Nassehi et al., 2016; Gao et al., 2018). Moreover, although no study has directly tested the association between perceived discrimination and problematic mobile phone use, the deleterious effect of perceived discrimination on other additive behaviors, such as substance abuse, has been illustrated (Gibbons et al., 2004; Pascoe and Smart Richman, 2009; Tran et al., 2010). Thus, it is reasonable to postulate that perceived discrimination could promote a higher level of problematic mobile phone use.

Furthermore, two studies have elucidated the role of perceived discrimination as a mediator in the association between stressful events and negative outcomes. Wagner et al. (2012) demonstrated that discrimination could be a key mediator underlying the association between posttraumatic stress and HIV treatment adherence. Bao et al. (2016) reported that family risk could impact sleep disorder through perceived discrimination. Based on existing evidence, we hypothesized that perceived discrimination would work as a mediator between parental rejection and problematic mobile phone use.

### School Engagement as the Mediator

School engagement has been defined as a multifaceted construct, including investment and participation in academic activities, identification with positive school-related outcomes, and strategic or self-regulated learning (Jimerson et al., 2003; Wang et al., 2011). Given that acceptance, warmth, supervision, and support from the family may be internalized and have an impact on future adaption in school contexts (Connell and Wellborn, 1991), school engagement as a malleable state could be shaped by family context (Annunziata et al., 2006; Smalls, 2009; Wang and Eccles, 2012). Adolescents with supportive and warm parents are inclined to show higher school engagement and better school performance (Li et al., 2010). By contrast, parental rejection, as a parenting style that lacks parental warmth, support, and other positive expressions (Rohner et al., 2005), may ultimately exert a negative effect on school engagement. In sum, an undesirable parent–adolescent relationship will restrict the development of school engagement (Zhu et al., 2015).

Although several studies have documented that decreased school engagement predicts lower school adjustment and more behavioral problems (Simons-Morton and Chen, 2009; Chase et al., 2014; Wang and Fredricks, 2014; Snyder and Smith, 2015), no study has tested the impact of school engagement on problematic mobile phone use. The current study addressed this gap and further tested whether school engagement could be a mediator between parental rejection and problematic mobile phone use. Previous studies delineating the relationship between school engagement and addictive behavior (e.g., Internet addiction, substance abuse) provide empirical support for the assumption that low school engagement is associated with problematic mobile phone use. For instance, in a longitudinal sample, Wang and Fredricks (2014) found that youth with lower school engagement showed higher substance use as well as delinquency over time.

Because mobile phones are in some cases portable Internet devices and thus often used by persons addicted to the Internet, some researchers have pointed out that mobile phone addiction is essentially similar to Internet addiction (Eduardo et al., 2012; Han et al., 2017). Evidence showing the predictive effect of lower school engagement on higher Internet addiction (Li et al., 2013; Zhu et al., 2015) suggests that there is an association between school engagement and problematic mobile phone use.

Moreover, Upadyaya and Salmela-Aro (2013) reviewed the associations among different social contexts, school engagement and youth adaption, and asserted that family context could have an impact on youth adaption through school engagement. Empirical studies have also demonstrated that school engagement mediated the association between family factors (e.g., parenting style, parent–child relationship) and individual development (Li et al., 2010; Zhu et al., 2015). Thus, in the current study, we hypothesized that school engagement would play a mediating role in the association between parental rejection and problematic mobile phone use.

Meanwhile, prior research has indicated that perceived discrimination could negatively influence school engagement (Smalls et al., 2007; Dotterer et al., 2009; Brody et al., 2012; Benner and Graham, 2013). Using a three-wave longitudinal design, Brody et al. (2012) found that youth who experienced higher discrimination reported more negative beliefs about the usefulness of schools, lower academic efficacy, and poorer academic achievement, which in turn led to decreases in school engagement. Another study in a sample of 148 African American adolescents demonstrated that racial discrimination could impede school self-esteem and school bonding (Dotterer et al., 2009). Hence, the current study assumed that the association between parental rejection and problematic mobile phone use would be mediated by both perceived discrimination and school engagement sequentially, such that perceived discrimination and school engagement may work together in a chain mediation model.

### The Current Study

**FIGURE 1:**
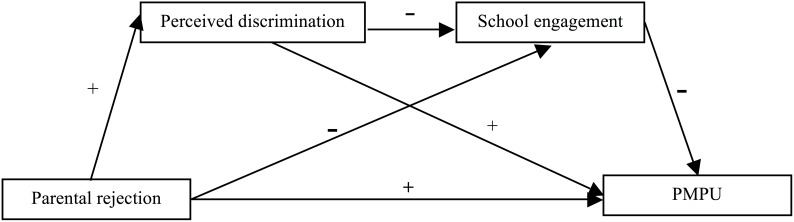
Proposed mechanism of the association between parental rejection and PMPU.

This study tested the direct effect of parental rejection on problematic mobile phone use as well as the role of two parallel and sequential mediation mechanisms in this association (Figure 1). Based on existing theoretical perspectives and empirical evidence, we hypothesized that in our sample of Chinese university students: (a) parental rejection would be positively associated with problematic mobile phone; (b) perceived discrimination would mediate the association between parental rejection and problematic mobile phone use; (c) school engagement would mediate the association between parental rejection and problematic mobile phone use; and (d) perceived discrimination and school engagement would be a sequential mediating mechanism in the association between parental rejection and problematic mobile phone use.

## Materials and Methods

### Participants

We recruited participants from three universities in the southern Chinese province of Guangdong. The sample consisted of 356 university students (36.3% male) ranging from 17 to 19 years of age (mean age = 18.33, *SD* = 0.57). Reflecting the demographics of the area, 30.0% came from rural areas, 13.8% from county seats, 27.6% from small-medium cities, and 28.5% from metropolitan areas. Moreover, 75.1% of participants’ fathers and 69.6% of their mothers had less than a junior college education. The average monthly income in 43.3% of recruited families exceeded ¥3000, which represents higher than average personal monthly household income (¥2857) in China (2015).

### Measures

#### Parental Rejection

The measure of parental rejection has been shown to have strong reliability and construct validity (Gerlsma et al., 1992; Gerlsma and Hale, 1997). Respondents were asked to indicate their experiences of parental rejection when they were growing up (e.g., “My parents are very critical of me”; “My parents get annoyed when I want something from them”; “My parents try to change who I am”). All items were rated on a five-point scale ranging from 1 (*never*) to 5 (*frequently recurring*). The responses were averaged across these three items, with higher scores indicating higher levels of parental rejection. In the current study, the Cronbach’s alpha was 0.76.

#### Perceived Discrimination

Perceived discrimination was measured by the nine-item discrimination questionnaire developed by Lee and Ferraro (2009). Respondents were asked to indicate their perception of discriminatory experiences on a daily basis (e.g., “Are you treated with less courtesy than other people?”; “Are you treated with less respect than other people?”; “Do you receive poorer service than other people at restaurants or stores?”). All items were rated on a five-point scale ranging from 1 (*never*) to 5 (*frequently recurring*). Item scores were averaged to create a composite score for perceived discrimination, with higher scores indicating higher levels of perceived discrimination. In the current study, the Cronbach’s alpha was 0.88.

#### School Engagement

School engagement was measured by the 23-item School Engagement Scale, which was originally developed by Wang et al. (2011). Respondents were asked to describe themselves in terms of the behavioral, emotional and cognitive components of school engagement (e.g., “How often have you skipped class?”; “In general, I feel like a real part in this school”). All items were rated on a five-point scale ranging from 1 (*never*) to 5 (*frequently recurring*). Item scores were averaged to create a composite score for school engagement, with higher scores indicating higher levels of school engagement. In the current study, the Cronbach’s alpha was 0.86.

#### Problematic Mobile Phone Use

Problematic mobile phone use was assessed using the 17-item Mobile Phone Addiction Index (MPAI; Leung, 2008). Respondents were asked to indicate how often they are bothered by problematic mobile phone use (e.g., “It is difficult for you to turn the mobile phone off”; “You will be upset if your phone is not available”). All items were rated on a five-point scale ranging from 1 (*never*) to 5 (*frequently recurring*). Item scores were averaged to create a composite score, with higher scores indicating higher levels of problematic mobile phone use. In the current study, the Cronbach’s alpha was 0.87.

#### Covariates

We controlled for gender, age, and SES as covariates in statistical analyses. Gender was a dichotomous variable (1 = male; 0 = female). Age was measured by the respondent’ s age in years. SES was measured as the average of a respondent’s standardized scores on four items (e.g., geographical area, educational level of each parent, family per capita monthly income). Respondents were asked to indicate the type of geographical area on a five-point scale (1 = *rural area*, 4 = *metropolis*) that ranged from less-developed to highly developed. Educational level of each parent was measured on a six-point scale (1 = *less than or equal to elementary school level*, 6 = *graduate level*). Income was measured by family per capita monthly income, a seven-category variable (1 = *less than or equal to ¥190*, 7 = *greater than or equal to ¥3000*).

### Procedure

Permission to implement the study was granted by the research ethics committee of corresponding author’s university. Verbal consent was obtained from participants. The parents of 14 participants who were younger than age 18 also provided consent. Trained researchers administered the self-report questionnaires to students during class time. The anonymity of the participants’ responses was emphasized at the beginning of the data collection session. Participants were also told that they must respond to the questionnaire items by themselves, and that they were free to withdraw at any time during data collection without penalty. The students received partial course credit for participating.

### Statistical Analysis

We estimated mediation effects using structural equation modeling (SEM) methods. Models were estimated using Mplus Version 7.0 (Muthén and Muthén, 2012), adopting the full information maximum-likelihood estimation procedure to accommodate missing data. A bootstrapping procedure was used to test the statistical significance of the paths and indirect effects in each model (Erceg-Hurn and Mirosevich, 2008). Model fit was assessed using multiple fit indices including the ratio of chi-square to degrees of freedom (*x^2^/df*), comparative fit index (CFI), root-mean-square error of approximation (RMSEA), and Tucker–Lewis index (TLI). The SEM literature shows that model fit is good when *x^2^/df* ≤ 3; CFI ≥ 0.95, TLI ≥ 0.95, and RMSEA ≤ 0.06 (Kline, 2011; Hoyle, 2012).

## Results

### Descriptive Statistics

85.9% of the 356 participants could be identified as problematic mobile phone uses. Means, *SD*s, and correlations of major study variables are displayed in Table 1. All the major study variables were significantly inter-correlated. Parental rejection was positively associated with perceived discrimination (*r* = 36, *p* < 0.01) as well as problematic mobile phone use (*r* = 0.21, *p* < 0.01), and was negatively associated with school engagement (*r* = -0.18, *p* < 0.01). Perceived discrimination was positively associated with problematic mobile phone use (*r* = 0.28, *p* < 0.01) and was negatively associated with school engagement (*r* = -0.32, *p* < 0.01). In addition, school engagement was negatively associated with problematic mobile phone use (*r* = -0.23, *p* < 0.01).

**Table 1:** Descriptive statistics and correlations among major variables (*N* = 356).

Variables	*M*	*SD*	1	2	3
1. Parental rejection	2.06	0.74	1		
2. Perceived discrimination	1.77	0.55	0.36**	1	
3. School engagement	3.78	0.43	–0.18**	–0.32**	1
4. Problematic mobile phone use	2.51	0.64	0.21**	0.28**	–0.23**


*^∗∗^p < 0.01.*

### Mediation Model Test

**FIGURE 2:**
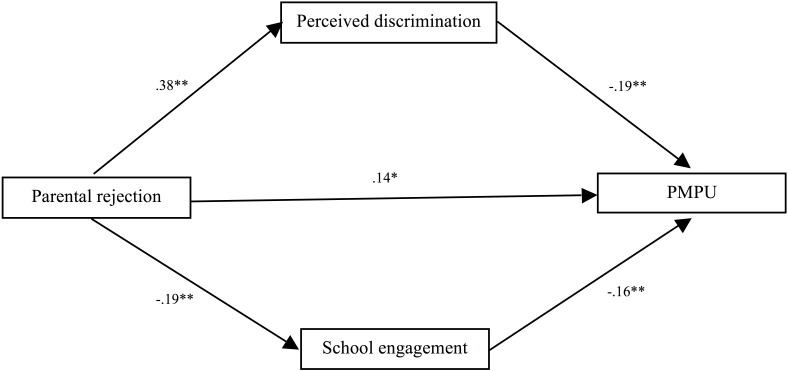
Tests of Model 1 showed parallel indirect effects of perceived discrimination and school engagement in the association between parental rejection and problematic mobile phone use. *Note.* Although not displayed for reasons of clarity, this model also included paths among controlled variables (i.e., age, gender, SES) and each of the variables in the model. PMPU = problematic mobile phone use.

We found that parental rejection was positively associated with problematic mobile phone use (*b* = 0.21, *p* < 0.001) before accounting for the mediation variables. Then, we followed a stepwise method to construct the best fitting model for the mediated effects of perceived discrimination and school engagement. First, we evaluated the fit of the parallel mediation model (Model 1) which included: (a) the direct path from parental rejection → problematic mobile phone use, (b) the indirect path from parental rejection → perceived discrimination → problematic mobile phone use, and (c) the indirect path from parental rejection → school engagement → problematic mobile phone use. In this model, all the paths were significant (see Figure 2) but the data did not fit the data well (i.e., *χ^2^/df* = 18.46, CFI = 0.88, RMSEA = 0.23, and SRMR = 0.03), implying that the parallel mediation model may be not the best model.

**FIGURE 3:**
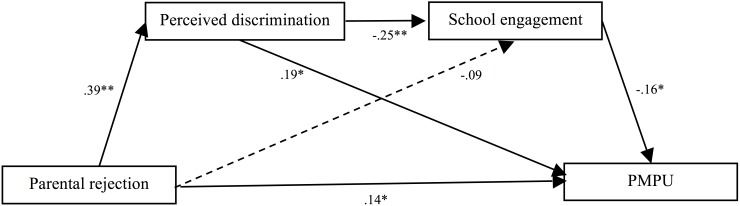
Tests of Model 2 showed the indirect effect of perceived discrimination, and the sequential indirect effects of perceived discrimination and school engagement, in the association between parental rejection and problematic mobile phone use. School engagement, when considered alone, did not act as a mediator. *Note.* Although not displayed for reasons of clarity, tests of this model also included paths among controlled variables (i.e., age, gender, SES) and each of the variables in the model. PMPU = problematic mobile phone use.

Second, we added the path from perceived discrimination to school engagement (Model 2, see Figure 3). This model was fully saturated (i.e., *χ^2^/df* = 0.00, CFI = 1.00, RMSEA = 0.00, and SRMR = 0.00). All the paths in this model were significant except that the association between parental rejection and school engagement did not hold (*b* = -0.09, *p* > 0.05). That is to say, perceived discrimination fully mediated the impact of parental rejection on school engagement.

**FIGURE 4:**
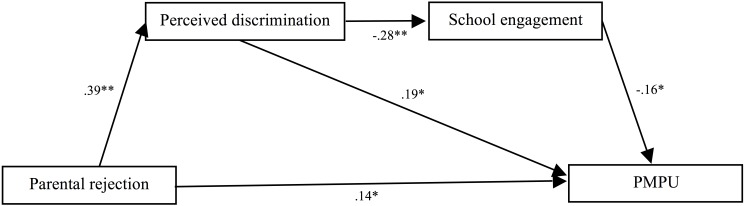
Tests of Model 3 showed that after removing the non-significant path between parental rejection and school engagement, there was an indirect effect of perceived discrimination, and sequential indirect effects of perceived discrimination and school engagement, in the association between parental rejection and problematic mobile phone use. *Note.* Although not displayed for reasons of clarity, tests of this model also included paths among controlled variables (i.e., age, gender, SES) and each of the variables in the model. PMPU = problematic mobile phone use.

Next, given that the saturated model (Model 2) was of little use statistically, we dropped the non-significant paths from parental rejection to school engagement (Model 3, see Figure 4). Model 3 showed a good fit, *χ^2^/df* = 2.67, CFI = 0.99, RMSEA = 0.07, and SRMR = 0.01, and it did not significantly decrease the model fit, *Δχ^2^*(1) = 2.67, *p* > 0.05. Therefore, Model 3 was the final mediated model in which the association between parental rejection and problematic phone use was mediated not just by perceived discrimination, but also by perceived discrimination and school engagement in sequence.

Finally, the indirect effects are reported in Table 2. Bootstrapping analyses indicated that the indirect effect of parental rejection on problematic mobile phone use through perceived discrimination was significant and positive (*beta* = 0.07, *p* < 0.01, and 99% CI [0.010,0.160]); and the indirect effect of parental rejection on problematic mobile phone use through both perceived discrimination and school engagement in sequence was significant and positive (*beta* = 0.02, *p* < 0.01, and 99% CI [0.001,0.042]). Additionally, the indirect effect of parental rejection on school engagement via perceived discrimination was significant and negative (*beta* = -0.11, *p* < 0.01, and 99% CI [-0.199, -0.051]). Perceived discrimination appeared to exert an indirect effect on problematic mobile phone use via school engagement (*beta* = 0.04, *p* < 0.01, and 99% CI [0.010,0.094]).

**Table 2:** Indirect effects in the final model.

Pathways	*beta*	99% CI
Parental rejection? perceived discrimination→ PMPU	0.07	0.024,0.141
Parental rejection→ perceived discrimination→ school engagement	–0.11	–0.199, –0.051
Perceived discrimination→ school engagement → PMPU	0.04	0.010,0.094
Parental rejection→ perceived discrimination→ school engagement→ PMPU	0.02	0.001,0.042
PMPU = problematic mobile phone use.		


## Discussion

The objective of this study was to test the association between parental rejection and problematic mobile phone use, as well as the explanatory mechanisms of this association, in a sample of Chinese university students. As expected, we found that parental rejection was a risk factor for problematic mobile phone use. Tests of mediation showed that students’ perceived discrimination might partly explain this risk process. That is, students who experienced more parental rejection were also more likely to perceive that they were discriminated against, leading to more problematic phone use. School engagement appears to be important also, but to a lesser degree. Although school engagement did not act as a mediator when considered alone, there was a sequential mediating effect in which parental rejection predicted perceived discrimination, which in turn predicted school engagement, which in turn predicted problematic mobile phone use. Next, we discussed each of our research questions in light of this multiple mediation model.

### The Direct Association Between Parental Rejection and Problematic Mobile Phone Use

Notably, parental rejection was positively associated with a higher level of problematic mobile phone use. This implies that university students who experience parental rejection may be more inclined to become problematic mobile phone users. This result adds to previous studies that have suggested that parental rejection enhances the risk for maladaptive development, from delinquency to psychopathology (Campo and Rohner, 1992; Miranda et al., 2016; Ramírez-Uclés et al., 2018). Consistent with problem behavior theory (Jessor, 1987), parental rejection may damage the perceived environment and elicit more problematic mobile phone use. Parental rejection could undermine undergraduates’ feelings of relatedness to their parents, and further lead to deficits in social competence (Dwairy, 2010). These students may avoid face to face interaction, and in turn select mobile phones to meet the need for communication with others on account of limited social resources and support. However, when mobile phone use helps people alleviate psychological imbalance due to parental rejection, it also drags them into another trap of excessive and unregulated mobile phone use (Kim et al., 2015).

### The Indirect Role of Perceived Discrimination

The current study also identified the role of perceived discrimination as a mediator in the link between parental rejection and problematic mobile phone use. Specifically, parental rejection was positively associated with perceived discrimination, which in turn predicted a higher level of problematic mobile phone use. According to parental acceptance–rejection theory (Rohner, 2004), self-esteem, self-adequacy, worldview, and emotional stability tend to be negatively impacted by parental rejection. On one hand, university students with a higher level of parental rejection may evaluate themselves negatively and internalize a more hostile world view because of impaired self-recognition (Ramírez-Uclés et al., 2018). The process of identity development is one of the major psychosocial tasks of late adolescence (McLean, 2005). Those late adolescents with the experience of parental rejection may be engaged into damaged process of self-definition and fail to forge a sense of identity in the context of previous adversity, leading to low self-esteem (Kenny and Rice, 1995). Those negative thoughts and feelings make them vulnerable to others’ negative attitudes toward them, and they then perceive a higher level of discrimination. On the other hand, parental rejection may lead to deficits in social skills and in emotion regulation (Meesters and Muris, 2004; Dwairy, 2010). Late adolescence is a time of social reorganization. Those university students move away form home, and then their peers or other adults come to meet their need for emotional support. Poor interpersonal problem solving caused by parental rejection may lead them easily to be the object of discrimination during the shift in attachment figures.

Furthermore, our findings confirmed the second path of the indirect effect: that perceived discrimination is a risk factor for problematic mobile phone use. This finding is consistent with previous research with regard to the influence of perceived discrimination on addictive behavior (Okamoto et al., 2009; Chia-Chen Chen et al., 2014). Perceived discrimination as a developmental risk could lead to a series of stress responses (McClure et al., 2010; Romero et al., 2014). Prior studies have highlighted that the use of communication devices or technology is useful to relieve stress and negative emotional states (Akin and Iskender, 2011; Tang et al., 2014; Jun and Choi, 2015). For example, Chiu (2014) reported that in a sample of university students, mobile phone use was useful in relieving negative emotions and experiences caused by interpersonal relationship stress. To advance this research, the current study provided evidence that people may use mobile phones excessively to tackle perceived discrimination, which is rooted in parental rejection. The current study is the first to underscore the mediation mechanism underlying the association between parental rejection and problematic mobile phone use.

### The Sequential Mediation Mechanism of Perceived Discrimination and School Engagement

The final structural equation model indicated that perceived discrimination was negatively associated with school engagement. This result is consistent with prior research reporting that perceived discrimination decreases youths’ school engagement and academic performance (Smalls et al., 2007; Dotterer et al., 2009; Brody et al., 2012; Benner and Graham, 2013). Brody et al. (2012) suggested that perceived discrimination would lead to feelings of devaluation and demoralization, which could cause students to become less inclined to take on conventional values and pursuits. This process may make students who have experienced discrimination view school as a useless and irrelevant social organization, further diminishing their belief in the importance of academics and the value of studying at school, their school-related self-esteem, and their educational aspirations (Thames et al., 2013; Unnever et al., 2016). In addition, perceived discrimination fully mediated the impact of parental rejection on school engagement. The insignificant association between parental rejection and school engagement implies that relative to parental rejection, perceived discrimination plays a more proximal and pronounced role in the prediction of school engagement. It is reasonable because perceived discrimination may interfere with identity development, which is a major task for late adolescents (Kenny and Rice, 1995). And then this identity-related dysfunction is likely to further affect individual development (e.g., decreased school engagement). Moreover, students in our study have moved away from family to university, and this shift could decrease the impact of parents on school activity. These results highlight the importance of testing the role of perceived discrimination in shaping student adjustment in the context of risk.

Moreover, we found that increased school engagement was significantly associated with less problematic mobile phone use. Several studies have reported the protective role of school engagement on maladaptive behavior (Li et al., 2013; Wang and Fredricks, 2014; Zhu et al., 2015). In alignment with social control theory (Hirschi, 1969), adolescents with higher school engagement are more likely to learn and to meet conventional expectations; hence, they may show a lower level of problematic mobile phone use. Furthermore, the present study found that school engagement worked as a mediator in the relationship between perceived discrimination and mobile phone addiction. Because of this process, a sequential mediation path appeared. That is, parental rejection could influence problematic mobile phone use through both perceived discrimination and school engagement. University students who experience parental rejection, which primes them to perceive more discrimination, may be inclined to become less engaged at school and ultimately become a problematic mobile phone user. This study integrates family, cognitive, and school factors that directly and indirectly influence problematic mobile phone use.

### Limitation

Some important limitations of this study should be noted when considering the findings. First, the data were collected through self-report, which may increase the shared method variance and limit objectivity. Although Rohner et al. (2009) suggested that children’s reports of parenting are more reliable than parents’ reports, it is still necessary for future research to use multiple methods (e.g., multiple informants, interviews, observations) to obtain a more objective index. Second, this study used a cross-sectional design. Although the final model contributes to our understanding of the factors that may influence problematic mobile phone use, it cannot verify temporal change or allow causal inferences. Some theoretical frameworks have suggested that there may be a reciprocal process in the association between parenting and children’s behavior (Patterson, 1982; Reid et al., 2002). Future research should adopt longitudinal designs and use cross-lagged models with all data obtained from all measures at all time points to test the directions of paths (Cole and Maxwell, 2003). Third, the current study did not test the mechanism by which parental rejection is associated with mobile phone usage time and different types of usage behavior. Usage behavior does not necessarily produce addiction indicators (Hong et al., 2012). To achieve a comprehensive understanding of our model, it is necessary to pay more attention to understanding the predictors of mobile phone usage time and usage behavior in the future. Last, we only tested the impact of parental rejection as a risk factor for problematic mobile phone use in a sample of university students. In fact, mobile phones have gradually become an essential part of life for much younger children and adolescents. Vernon et al. (2018) reported that mobile phone use was directly associated with increased externalizing behavior and decreased self-esteem in a sample of adolescents aged between 13 and 16 years old. Thus, it is also important to discuss and compare the effect of parental rejection on problematic mobile phone use among people in different developmental periods. Finally, research is needed to replicate our results in more diverse samples.

## Conclusion

The study shows the roles of perceived discrimination and school engagement in shaping problematic mobile phone use among university students who experience parental rejection. Specifically, perceived discrimination and school engagement can exert sequential mediating effects on the path between parental rejection and problematic mobile phone use. The results reveal the onset mechanism of problematic mobile phone use from the perspectives of parenting, personality, and school, and also provide empirical support for the association between parenting and problematic mobile phone use. It is hoped that these results will have applied value in preventing and treating problematic mobile phone use by reducing university students’ perceived discrimination and increasing their school engagement.

## Ethics Statement

This study was carried out in accordance with the recommendations of the Research Ethics Committee at South China Normal University guidelines, the Research Ethics Committee at South China Normal University with written informed consent from all subjects. All subjects gave written informed consent in accordance with the Declaration of Helsinki. The protocol was approved by the Research Ethics Committee at South China Normal University.

## Author Contributions

JZ, RX, and WZ conceived and designed the research. RX and JZ performed the research. JZ, RX, and YC analyzed the data. JZ, RX, YC, and WZ contributed to the writing of the manuscript.

## Conflict of Interest Statement

The authors declare that the research was conducted in the absence of any commercial or financial relationships that could be construed as a potential conflict of interest.
